# Supplementary material for: Are carbon emissions trading and green financial instruments synergistic? -Comprehensive quantitative research based on content analysis
Source: PLoS One. 2024 Mar 7;19(3):e0298601. doi: 10.1371/journal.pone.0298601 (PMC10919601; doi:10.1371/journal.pone.0298601)
Supplement: S1 Data — (ZIP) [file pone.0298601.s001.zip › Data/地级市Z_F工作报告.zip!/║■▒▒/╬Σ║║╩╨/└┤╫╘╣1⁄2╓┌║┼í╛┬φ┐╦╩2╛▌═°í┐_2016.docx]

各位代表：

现在，我代表市人民政府向大会报告工作，请连同《武汉市国民经济和社会发展第十三个五年规划纲要(草案)》一并审议，并请市政协各位委员和其他列席人员提出意见。

一、2015年和“十二五”工作回顾

2015年，我们在省委、省政府和市委的坚强领导下，积极适应引领经济新常态，抢抓机遇、主动作为，克难攻坚、砥砺奋进，在建设国家中心城市、复兴大武汉的伟大征途上阔步前进。这一年，我们在困难和挑战中稳定增长，主要经济指标位居同类城市前列，“万亿倍增”开局良好；这一年，我们在动力转换中加快创新，国家创新型城市建设热潮涌动，创新创业蔚然成风；这一年，我们在深化改革中破解难题，重点领域率先突破，制度红利加速释放；这一年，我们在完善功能中提升品质，“绿道年、路网年、停车场年”建设成效显著，城市面貌日新月异；这一年，我们在加快发展中改善民生，社会建设全面加强，市民福祉不断增进。经过一年艰苦努力，我们较好完成了市十三届人大四次会议确定的主要目标任务，为“十二五”发展画上了圆满句号，为“十三五”开局打下了坚实基础。

预计地区生产总值增长8.8%左右；地方一般公共预算收入1245亿元，增长12%；全社会固定资产投资增长10%以上；社会消费品零售总额增长11.5%左右；城镇常住居民人均可支配收入增长9%左右，农村常住居民人均可支配收入增长10%左右。

过去的一年，我们主要抓了以下几个方面的工作：

一是突出服务企业、项目支撑，全力以赴稳增长。及时出台支持企业发展“1+5”政策措施。建立重大产业和城建项目周末调度、每月十企座谈等制度，开展“万名干部进万企、创优服务稳增长”等主题活动，政企联动、共渡难关。

高端优质项目呈现“多点齐发”新局面。投资50亿元以上工业项目开工8个、投产2个，投资10亿-50亿元工业项目开工52个、投产49个，完成工业投资2769亿元。上汽通用武汉基地实现当年达产，联想摩托罗拉项目实现量产，力诺新康化工产业园、长飞科技园一期等建成投产，华星光电、天马LTPS 6代线、比亚迪新能源汽车等项目加快推进。永旺经开梦乐城、宜家荟聚购物中心等重大服务业项目开业。

经济运行呈现“四个强劲”新亮点。重点工业行业增势强劲，汽车及零部件、电子信息产业产值分别达2614.5亿元、1780.2亿元，增长22.3%、11.2%。金融业增势强劲，存贷款余额分别增长16.1%、18.4%。房地产业增势强劲，新建住房销售22.5万套、2283.8万平方米，均居全国城市第2位，分别增长25.1%、27.1%。新兴商业业态增势强劲，电子商务交易额达4135亿元，增长33.5%。

结构调整呈现“两升两降”新态势。高新技术产业比重升，占规模以上工业总产值比重提高2.7个百分点，重化工业比重降，占比下降3.2个百分点；工业生产升、单位能耗降，规模以上工业增加值增长8.5%，单位能耗下降9.5%左右。服务业结构持续升级，“互联网+”产业创新工程率先发力，新增泰康人寿在线保险等122家互联网金融企业，长江大数据交易所(筹)、东湖大数据交易中心成立运营，互联网企业、软件和信息服务业迅猛发展；社会物流总额2.8万亿元，工程设计、旅游等产业收入保持两位数增长。

二是突出创新引领、动力转换，推进国家创新型城市建设。研究制定支持创新“1+9”政策。加快提升创新能力。新建工程技术中心、企业研发中心等各类科技创新平台139个，新引进世界500强、中国500强和跨国公司研发机构23个，引进海内外高层次创新人才390名。发明专利授权量6003件，增长55%。

创新科技体制机制。在全国率先开展科技金融改革创新试验。全面推进职务科技成果使用、处置和收益管理改革，武汉地区10所试点高校共转移转化科技成果5372项。新三板挂牌企业新增70家，累计149家。

大力发展创新型经济。5个专业园区纳入东湖国家自主创新示范区“一区多园”试点。新建科技孵化器116万平方米，新认定国家级众创空间14家，全市孵化器、众创空间、大学生创业特区新创办企业1822家。高新技术产业产值达7700亿元。

三是突出功能提升、品质改善，全面协调推进城市建设。树立“让城市安静下来”理念,突出抓好城市建设。加快推进重大交通枢纽项目。阳逻港集装箱三期开港运行，天河机场第二跑道、机场交通中心主体工程完工，机场旅客吞吐量1894.2万人次，居中部第一。光谷火车站开工建设。获批国家综合运输服务示范城市。宽带网络提速降费。

完善路网体系。首条穿越汉江地铁——3号线一期开通试运营，地铁成环、通达三镇。二环线全面贯通，东湖隧道、江汉六桥、中法友谊大桥建成通车，三环线、中南路、中北路、21号公路、青王公路、长丰大道等完成改造。轨道交通5号线、1号线径河延长线、蔡甸线开工建设，11条地铁线同时建设。新开工汉江大道、青山长江大桥等项目。新改建次支路130条，建成慢行交通系统160公里，新增停车泊位16210个。

实施排涝治污供水攻坚行动计划。新增排水干网109公里、污水管网110公里、供水管道30.5公里，完成三金潭、汤逊湖、黄浦路一期等污水处理厂改扩建，余家头、琴断口自来水厂改扩建工程完工。获批开展全国海绵城市试点。

加强环境治理生态保护。园博园建成开放，成为城市生态新地标，金口垃圾填埋场生态修复项目荣获联合国气候变化大会C40城市奖。东湖绿道启动建设，环汉口绿道、环墨水湖绿道加快推进，新增绿道233.7公里。四环线生态林带开工建设，张公堤城市森林公园建成开放，修复破损山体4333.5亩。智慧湖泊管理系统上线试运行。集贸市场、城乡结合部等薄弱区域环境治理不断加强，建成长山口垃圾焚烧厂二期工程，新建地埋式垃圾收集站97座，新改扩建公厕117座。

四是突出问题导向、关键环节，蹄疾步稳推进改革攻坚。加大简政放权力度。市级权力事项由4516项压缩到1822项，市级行政审批事项由240项减少到166项，为全国审批事项较少城市；全面实行工商注册“三证合一”登记制度；东湖新技术开发区、武汉经济技术开发区率先开展“行政审批3.0”改革，实现“一枚印章管审批”。坚持依法行政，出台《武汉市人民政府重大行政决策程序规定》及系列配套文件；认真贯彻新《行政诉讼法》，制定实施行政负责人出庭应诉新规。提请市人大常委会审议地方性法规草案7件，制定政府规章9件，人大议案、代表建议和政协建议案、提案全部办结。

加快发展民营经济。制定非公企业进入特许经营领域实施办法，积极推进城建PPP项目。私营企业达28.3万户，增长20.9%。

推进社会治理创新。出台创新社会治理加强基层建设“1+10”系列改革举措。100个老旧社区“幸福社区”创建工作全面完成，85%的住宅小区组建业主委员会。编制《武汉市社会信用体系建设规划》。推进阳光信访、法治信访、责任信访。

五是突出开放合作、拓展视野，提升国际化水平。新引进投资50亿元以上工业项目12个、30亿元以上服务业项目10个，新引进世界500强企业14家。实际利用外资73.4亿美元，增长18.5%。新开通武汉至澳大利亚黄金海岸、意大利罗马等6条国际直达航线，武汉成为中部首个72小时过境免签城市。中欧(武汉)国际货运班列回程货量居全国中欧班列第一。第十届中国(武汉)国际园林博览会精彩开幕。英国驻汉总领事馆开馆，香港特别行政区政府驻武汉经贸办事处设立。

主动融入长江经济带建设。在武汉设立长江中游城市群合作秘书处，四省会城市联合推出“双创”合作一体化政策，实现医保异地就医及时结算。对口援建帮扶工作扎实开展。

六是突出城乡统筹、转型示范，提高农村农业发展质量。创建国家现代农业示范区。提档升级7万亩设施蔬菜基地，推广肉类蔬菜二维码追溯系统，“菜篮子”综合自给率超过70%。农产品加工业产值达2510亿元。各类新型农业经营主体达1.35万家，新一轮农村土地承包经营权确权登记颁证基本完成。

改善农村生产生活条件。完成农村排灌港渠整治3506公里。推进农村饮水安全建设工程，惠及300个村22.5万人。全市行政村实现村村通客运班车。开展农村环境整治，35%的行政村环境卫生达标。推进精准扶贫，脱贫9362人。

七是突出以人为本、惠民利民，着力提高市民生活水平。全市公共财政用于民生领域的支出达939亿元，增长17.8%。新增就业18.8万人，农村劳动力转移就业5.02万人。健全城乡居民、被征地农民基本养老保险制度，社会保险净增参保42.4万人次。建成保障性住房8.9万套。

加强文化建设。全面实施“三个千万人次”文化惠民工程。江汉关博物馆、武汉科技馆新馆建成开放。全民健身运动广泛开展，成功举办WTA武汉网球公开赛、世界中学生田径锦标赛、亚洲田径锦标赛等国际赛事。获得第七届世界军人运动会承办权。

大力发展社会事业。各类教育事业协调发展。完成40所公办幼儿园新改扩建、99所公办小学标准化建设任务。职业教育集团化发展和校企合作取得新成效。启动国家公立医院综合改革试点。组建707个全科医生服务团队，签约服务城乡居民240万人。出生人口性别比持续下降。

健全公共安全体系。完善立体化治安防控体系，近12年来，首次实现刑事警情同比下降，“六五”普法圆满完成。食品安全城市创建工作深入开展，安全生产形势保持平稳。

国家安全、国防动员、军民融合、双拥、民防、消防、仲裁、保密、参事、气象工作积极推进，文史、档案、民族、宗教、侨务、地方志工作得到加强，妇女、儿童、老龄、残疾人工作取得新成绩。

各位代表，2015年各项目标的实现，标志着我市“十二五”规划圆满收官。“十二五”以来，全市人民紧紧围绕建设国家中心城市、复兴大武汉的宏伟目标和武汉2049远景战略，敢为人先、追求卓越，经济社会发展取得重大成就，书写了无愧于时代的辉煌篇章!

这五年，经济总量实现历史性跨越，跃上万亿元台阶，进入全国城市第一方阵。工业倍增计划成效明显，工业投资累计10537亿元，形成大光谷、大车都、大临港、大临空四大工业板块，千亿元产业从1个增加到5个。80万吨乙烯、上汽通用武汉基地、联想武汉基地、东风本田二厂、神龙三厂等重大项目建成投产，“新武汉造”加速崛起。现代服务业快速发展，金融、物流、商贸、会展、工程设计、旅游等优势行业进一步壮大，5A级景区位居同类城市首位，文化创意、软件信息、电子商务等新兴产业发展迅猛。

这五年，城市建设规模空前，城市面貌发生根本性变化。地铁、快速路网、过江通道和城市有机更新改造引领城市蝶变，城建累计投入6870亿元，是“十一五”的2倍。地铁从无到有，连续4年每年开通一条线路，运营里程达126公里。中心城区快速路网基本形成，建成武汉大道、二七长江大桥、鹦鹉洲长江大桥，开工建设四环线。综合交通枢纽地位巩固提升，“米”字形高铁中心正在形成；武汉长江中游航运中心建设纳入国家战略，阳逻港迈入世界内河集装箱港口第一方阵。建成武汉市民之家、辛亥革命博物馆、光谷国际网球中心、武汉国际博览中心、楚河汉街等一批城市新地标。“城管革命”强力推进，城市环境显著改善。

这五年，改革创新先行先试，获批国家全面创新改革试验区，发展活力竞相迸发。全面深化改革，新一轮政府机构改革顺利完成，率先推出权力清单、责任清单、程序清单。开展电视问政，严格治庸问责。出台促进科技成果转化的“黄金十条”，高校职务科技成果“三权”改革走在全国前列。武汉获批国家知识产权示范城市。累计获国家科技进步特等奖4项、一等奖6项；高新技术企业达1529家，比“十一五”末净增1006家。

这五年，国际大通道加快形成，国际化水平显著提升，城市影响力不断增强。国际和地区航线新增30条，达37条，天河机场成为中部地区国际航线最多、国际国内旅客吞吐量最大的国际机场。开通至东盟、日本、韩国等近洋航线。中欧(武汉)国际货运班列开通并实现双向常态化运营。来汉投资的世界500强企业达230家。累计实际利用外资269.9亿美元、进出口总额1188亿美元，分别是“十一五”的2倍、1.93倍。

这五年，民生社会事业显著进步，城市文明建设跃上新高度，成功摘取全国文明城市桂冠。“好人之城”享誉全国，拥有全国道德模范11人，位居同类城市第一。民生福祉持续改善，社会保险扩面新增420万人次，保障性住房建设规模、教育均衡发展水平、医疗资源总量和服务能力均居全国同类城市前列。武汉被确定为全国少数民族服务管理示范城市。我市荣获全国社会治安综合治理最高奖“长安杯”，荣膺国家卫生城市称号。

各位代表!武汉，每天不一样!我们共同生活的这座城市，生机勃发、欣欣向荣。五年的成就，得益于党中央、国务院的方向引领和深切关怀，得益于省委、省政府的坚强领导和关心支持，得益于市委的科学谋划和总揽全局，凝聚着千万武汉人民的聪明才智和辛勤汗水，承载着历届市委、市政府打下的良好基础。在此，我代表市人民政府，向全市各族人民，向给予政府工作大力支持的人大代表、政协委员，向各民主党派、各人民团体和各界人士，向中央在汉单位和各类驻汉机构，向驻汉部队、武警官兵，向为武汉建设发展提供智力支持的专家学者，向参与城市建设的劳动者，向所有关心支持武汉建设发展的港澳台同胞、海外侨胞和国际友人，表示崇高敬意和衷心感谢!

在看到成绩的同时，我们也清醒地认识到武汉发展和政府工作中还存在着一些不足和短板：创新链不够完整，创新生态不够优化；工业总量还不够大，推动传统产业优化升级、战略性新兴产业发展壮大的步伐不够快。在大规模推进城市建设的同时，解决“城市病”突出问题还没有完全同步，交通拥堵还比较突出，排水系统建设滞后于城市发展，“摊大饼”现象还没有得到彻底遏制。城市管理不够精细，有的方面还比较粗放、甚至缺失，空气优良天数偏少，雾霾天气时有发生；保护水体、提升水质任务还很艰巨，保护湿地、山体等生态系统措施亟待加强。城乡居民收入水平与城市经济发展位次还不相适应。一些政府工作人员主动作为、勇于担当的精神还不够强。我们将正视矛盾、直面问题、弥补短板，让全市人民感受到实实在在的变化和成效!二、“十三五”发展基本思路和重点任务

“十三五”时期，是武汉率先全面建成小康社会的决胜阶段，是推进经济总量“万亿倍增”、建设国家中心城市的关键阶段，也是多重国家战略在武汉叠加的黄金机遇期。市政府根据《中共武汉市委关于制定国民经济和社会发展第十三个五年规划的建议》，编制了《武汉市国民经济和社会发展第十三个五年规划纲要(草案)》。

“十三五”时期，全市经济社会发展的总体思路和发展目标是：高举中国特色社会主义伟大旗帜，深入贯彻习近平总书记系列重要讲话精神，牢固树立创新、协调、绿色、开放、共享发展理念，坚持“四个全面”战略布局，坚持发展是第一要务，坚持“竞进提质、升级增效，以质为帅、量质兼取”工作方针，积极主动适应引领新常态，统筹推进经济建设、政治建设、文化建设、社会建设、生态文明建设，系统推进国家全面创新改革试验，加快建设具有全球影响力的产业创新中心，全力打造经济、城市、民生“三个升级版”，率先全面建成小康社会，巩固综合经济实力全国城市第一方阵地位，力争进入第一梯队，为建设国家中心城市、复兴大武汉奠定坚实基础。

为此，我们要践行“五大理念”，实现“五大提升”。

(一)坚持创新发展，加快转型升级，实现经济规模质量同步大提升

创新引领未来，发展在于创新。我们要加快创新型城市建设，培育发展新动能，构建产业新体系，打造武汉经济升级版。到2020年，全市地区生产总值达1.9万亿元。

系统推进全面创新改革试验，建设国家创新中心。实施产业创新能力倍增计划，以信息技术、生命健康、智能制造等三大领域为核心，构建以企业为主体，“产业链、创新链、人才链、资金链、政策链”五链统筹的产业创新生态体系。实施“城市合伙人”计划，与创新创业人才结成命运共同体、奋斗共同体，打造国际人才自由港。实施“创谷”计划，做实做强武汉未来科技城、融科智谷、岱家山科技创业园等创新园区，让众创空间遍布全城，掀起千军万马争创新、铺天盖地竞创业的热潮。到2020年，聚集全球创新要素的能力得到极大提升，东湖国家自主创新示范区基本建成世界一流科技园区，武汉建成国家创新型城市。

实施《武汉制造2025行动纲要》，建设国家先进制造业中心。实施智能制造工程，推动生产型制造业向生产服务型制造业转型，构建新型制造体系。实施战略性新兴产业倍增计划，建设国家存储器基地、新能源汽车产业基地、新型航天技术产业基地，发展新一代显示技术，壮大生物医药、高档数控机床等战略性新兴产业；推动汽车、石化、食品、家电等现有支柱产业向中高端升级，推动汉派服装产业转型升级；培育发展人工智能、无人机、3D打印、石墨烯材料、氢能等未来产业，构建“传统支柱产业—战略性新兴产业—未来产业”有机衔接、持续升级迭代的产业体系。武汉经济技术开发区建成全国一流的智能制造示范基地。武汉临空港经济技术开发区建成临空制造业聚集区。到2020年，高新技术产业产值达1.65万亿元。

实施现代服务业倍增计划，建设国家商贸物流中心。推动生产性服务业向专业化和价值链高端延伸、生活性服务业向精细化和高品质升级。鼓励传统商业转型升级，支持O2O、B2C、C2B等新型商业业态健康发展。突出科学定位、总部优先、功能提升，加快建设武汉中央文化区、武汉中央商务区、汉正街中央服务区、武汉国际博览会展区、汉口沿江商务区、青山滨江商务区、杨春湖高铁商务区等现代服务业聚集区。努力建成国家物流枢纽、商贸中心、中部金融中心、国家级会展中心，推进国家级旅游业改革创新先行区建设，创建国家旅游休闲示范城市、世界设计之都。

发挥市场和政府两个作用，构建发展新体制。加强供给侧结构性改革，提高全要素生产率，激发各类市场主体活力。加快发展科技型中小企业、创新型民营经济。深化国有企业改革，大力发展混合所有制经济。建设法治政府、服务型政府，“放管服”并重推进简政放权，从源头上杜绝各种“奇葩证明”，建立行政权力事项取消下放后的事中事后监管机制。推广“行政审批3.0”改革，推动设立区综合行政审批机构，变“多头受理”为“一口受理”。

高标准、高水平建设智慧城市，提高经济社会发展智能化水平。深入实施《武汉市智慧城市总体规划》。落实国家大数据战略，建设超级计算中心，增强大数据、云计算等城市服务功能。实施“互联网+”和物联网应用示范工程。实施智慧政府工程，建立以市民和企业为中心的政务公开服务体系，提供线下线上智能化、平台式服务。

(二)坚持协调发展，强化统筹兼顾，实现整体发展水平大提升

协调是持续健康发展的内在要求。我们要提高城市规划建设管理的协调性、系统性，统筹地上地下，统筹老城新区、城乡一体发展，增强城市发展整体水平。

加强规划、建设、管理相互衔接，实现空间规划与产业布局、城市功能协调统一。一座城市，既要看脸，也要看气质。我们要追求完美、注重细节、彰显品质、打造经典。

深化“1+6”城市空间格局体系规划。促进主城区、新城区、开发区(功能区)功能互补、良性互动。主城区严格控制在三环线以内，“三镇三城”均衡发展，全面完成三环线内城中村改造任务。新城区要规划先行、建设有序、生态优先、产城融合，加快形成规模适度、职住平衡、特色鲜明的现代化生态新城。全面划定城市开发边界，同时，在规划建设区注重留白，为城市未来发展留下空间。

巩固提升全国性综合交通枢纽地位。高铁瞄准“350”，推动建成武汉至西安高铁，推动规划建设沿江高铁和武汉至福州、武汉至杭州高铁，争取武汉至青岛、武汉至贵阳高铁纳入国家高铁中长期规划，全面形成“米”字形高铁网，打造全国高铁中心；建成光谷火车站，尽早启动建设新汉阳站，谋划建设高铁机场站。航运瞄准“645”，积极参与推动长江深水航道整治工程，初步建成长江中游航运中心，到2020年，港口集装箱年吞吐能力达500万标箱，多式联运、集疏运体系基本完备。打造重要门户枢纽机场，天河机场年旅客吞吐能力达4500万人次，谋划第二机场选址。高速公路总里程达790公里。

实施“畅通工程”。新建成13条轨道交通线，每年通2条地铁线，实现“主城联网、新城通线”，到2020年，轨道交通通车总里程达400公里，努力打造具有世界先进水平的“地铁城市”。建成环射成网、循环连通的城市快速路网体系，适当加密、规划建设连接二环三环、过江越湖快速通道，打造二环内30分钟畅通圈，建成四环线，推进外环东绕，打造市域快速交通圈。建成“公交都市”示范城市。

建设百年市政设施。采取“渗、滞、蓄、净、用、排”等措施，深入推进海绵城市建设；着力应对极端天气，切实提升排渍能力，加快解决城市渍水。推进雨污分流，完善污水全收集全处理系统。根据老城新区不同特点，科学规划建设地下综合管廊。强化能源供应保障，规划实施“特高压靠城、超高压进城”工程。统筹优化管道气、液化天然气、压缩天然气等各类燃气设施建设，加快新能源汽车充电桩建设。抓紧补齐建筑垃圾消纳、餐厨垃圾综合处理、危险固体废弃物规范管理等短板。推进城市管理升级，提升精细化、规范化、现代化管理水平。

推动城乡协调发展，提高农村农业发展水平。突出现代都市农业特色，率先转变农业发展方式，率先建立现代农业体系，率先实现农业现代化。建设国家现代农业示范区，加快国家新型城镇化试点，推进以人为核心的新型城镇化，新城区构筑中心区域明确、中心镇为支撑，生态小镇、新型社区蓬勃发展的现代城镇体系。全面实施脱贫攻坚工程，按照“精准扶贫、不落一人”的总要求，通过采取“五个一批”等措施，确保2018年现有扶贫对象全部脱贫。

深化文化五城建设，提升城市文化软实力。坚持用社会主义核心价值观凝聚共识、汇聚力量，建设全市人民共有精神家园。实施文明城市提升工程，增强武汉自信，强化认同感，增加获得感，共享荣誉感。

打造全国一流的文化功能区。建设琴台中央文化艺术中心，加快青岛路片历史街区保护改造，形成以琴台—龟北区域为一核，以武昌古城—环东湖沙湖和汉口沿江—张公堤园博园为两带的文化功能空间格局。

提升城市文化软实力。彰显知音文化、盘龙文化、木兰文化、首义文化等历史文化魅力。扩大琴台音乐节等文化名片的国际影响。实施重大文化活动、体育赛事培育工程，办好第七届世界军人运动会等一批重大国际赛事，打造文化体育休闲之都。面向不同群体，满足不同需求，重振汉派传统文化雄风，培育新潮文化汉军。建设全国重要的文化产业基地。

扩大城市影响。建设国际化交流平台，支持传统媒体与新媒体融合发展。开展“全球营销”，讲好武汉故事，树立武汉形象，传播武汉声音,提高城市知名度和美誉度，让武汉成为世界的武汉、令人向往的武汉!

(三)坚持绿色发展，建设美丽武汉，实现生态环境质量大提升

“万物各得其和以生,各得其养以成。”我们要把生态文明建设放在更加突出的战略位置，统筹生产生活生态，推进绿色富市、绿色惠民，保护好武汉的蓝天碧水净土。

优化城市生态格局。全面划定基本生态保护边界。对城市建设用地实行高效集约使用，对生态底线区实行严格保护。根据不同特点，分别对一环、二环、三环、外环、“六楔”和放射性道路，在现有基础上强化生态功能、提升绿化景观；同步建设四环生态林带。形成“两轴五环、六楔多廊、蓝绿织城”的多层次城市生态格局。

推进节能低碳循环发展。资源有限、循环无限，我们将实施循环发展引领计划，加强青山区等国家级循环经济园区试点和经验推广，加快工业园区循环化改造，构建循环型服务业体系。深化低碳城市试点，推动钢铁、化工、电力等重点行业节能控碳，发展低碳交通，推广绿色建筑，兑现碳排放达峰承诺。

加强生态环境保护。崇尚自然之美，让好山好水好风光融入城市。编制实施“拥抱蓝天”、“拥抱绿水青山”行动方案。实施城市蓝网工程，加快构建大东湖、金银湖等生态水网，中心城区、新城区建成区湖泊实现全部截污，全面建成三环线内湖泊公园。优化提升“两江四岸”江滩生态环境，打造百里滨江画廊。实施城市绿网工程，新建1300公里的绿道系统，编制实施全市446个山体保护规划，实现“绿满江城、花开三镇”。采取“八禁”和“推广、优化、转型、提升”系列措施，切实改善大气环境，实现优良天数逐年上升、污染天气逐年减少，基本消除内生原因产生的重污染天气。创建国家环保模范城市。

各位代表!绿色是万象之元，生态是万业之基。我们要像保护眼睛一样保护生态，像珍惜生命一样珍惜绿色，让大武汉呈现更多“高颜值”的碧水蓝天。

(四)坚持开放发展，拓展互利合作，实现国际化水平大提升

开放是繁荣发展的必由之路。探索内陆城市扩大开放新路径、新模式，构建全方位开放新格局、新优势，把武汉打造成面向世界的内陆开放高地。

主动对接国家战略，构建多层次开放格局。积极融入“一带一路”，建设中欧班列中部归集中心，拓展武汉至中国近海、东北亚、南亚等近海近洋航线。积极参与长江经济带建设，进一步强化长江中游城市群中心城市作用。努力把武汉打造成联络“一带一路”和长江经济带的重要枢纽城市。

加快发展开放型经济，提升经济外向度。推动外贸、外资、对外投资优化升级，努力建设内陆外资密集区、区域性国际贸易中心。2020年，进出口总额在2015年基础上实现翻番，在汉世界500强企业超过300家。

完善开放功能环境，建设中部国际化服务中心。实施口岸功能提升工程。积极争取设立湖北(武汉)内陆自贸区。建设中法武汉生态示范城。拓展国际大通道，国际直飞航线通达五大洲。加快外国领事馆区建设，争取更多国家在汉设立领事馆和办事机构。推行国际标准、规则、标识，优化国际化环境，办好各类国际展会。建设国际医院，增加国际学校和国际型社区。

(五)坚持共享发展，增进民生福祉，实现市民获得感幸福感大提升

民之所望，政之所向。我们要始终坚持以人民为中心，把政府工作的成就感建立在市民的获得感上，推进民生保障升级和社会治理水平提升，加快建设幸福武汉。

实施民生托底计划。出台更加积极的就业政策，建立面向大众的创业服务平台，五年城镇新增就业90万人以上，创业10万人以上。养老保险全覆盖、医疗保险补短板，建立更加公平更可持续的社会保障制度。创新公积金管理制度，支持保障性住房建设和多元化住房消费需求，完善保障性住房供应体系，实现低收入住房困难家庭应保尽保，创造条件让新市民实现“居者有其屋”。

实施公共服务供给创新计划。充分发挥市场作用，创新公共服务供给模式，不断满足多层次、普惠性和个性化服务需求。建设现代化教育名城，全市新增劳动力平均受教育年限提升到14.6年，率先实现教育现代化。推进健康城市建设，支持筹备建设质子重离子治疗中心，武汉地区卫生资源总量进入全国城市前三，医学高端人才、医学科技创新、居民健康水平等指标进入全国前列，建成国家医疗卫生服务中心。探索具有武汉特色的医养融合、智慧养老模式。完善人口发展战略，促进人口均衡发展。

实施社会治理能力提升计划。完善党委领导、政府主导、社会协同、公众参与、法治保障的社会治理体制，构建全民共建共享的社会治理格局。深化拓展网络化管理，推动街道(乡镇)成为基层发现和解决问题的中枢，完善社区(村)治理体系。突出“图之于未萌，虑之于未有。”完善社会治安立体防控体系，深化“平安武汉”建设。切实保障食品药品安全，构建现代超大城市防灾减灾体系，努力建设最安全城市。

各位代表，“凡是过去，皆为序章。”武汉的发展前后相续，今天的奋斗将成就明天的荣光。当历史的航船驶入2020，我们的城市，将成为一座产业高端、经济繁荣的实力之城，活力迸发、引领未来的创新之城，连通四海、融入世界的开放之城，文化先进、生态宜居的品质之城，成果共享、全面小康的幸福之城，率先向第二个百年目标进发。“为者常成，行者常至。”我们坚信，武汉的明天一定会更加美好!三、2016年工作建议

千里之行，始于足下。今年是实施“十三五”规划的第一年，新开局要有新气象，新起步要有新作为。

今年，全市经济社会发展的主要预期目标是：地区生产总值增长9%；地方一般公共预算收入增长10%；全社会固定资产投资增长12%，社会消费品零售总额增长12%；城乡居民收入增长与经济增长同步。全面完成省下达的节能减排任务。

我们将重点抓好以下几个方面的工作：

(一)加快建设国家创新型城市，在聚集创新要素、提升创新能力上取得新突破

启动全面创新改革试验，推进“五链统筹”，破解创新瓶颈，优化创新生态，形成一批可复制、可推广的经验。

完善以企业为主体的创新机制。制订实施大中型企业研发机构全覆盖行动计划，推动高新技术企业普遍建立研发中心。引进30家世界500强、中国500强企业和跨国公司研发机构。完善鼓励创新的政策体系。制定创新产品认定办法，扩大中小型科技企业创新产品和政府服务采购比例。深入推进科技金融改革创新试点，设立天使投资母基金。

全面推进创新园区建设。结合“三旧”改造、工业园区建设，编制创新园区空间布局规划，制定土地规划、人才奖励、天使投资等政策。着力引进一批有能力有经验投资建设、管理运营创新园区的大企业，创办一批智谷空间、创客空间等新型孵化器，建设一批创新园区。加快建设“大学之城”，打造环高校众创圈、大东湖创客带。推进汉口科技新城建设。

着力培育引进高端创新人才。积极实施“城市合伙人”计划，推进“3个10条”政策落地，建立全球产业领军人才信息库，力争引进不少于2名产业顶尖领军人才、200名企业高层次人才。实施摇篮工程，加快建设各类青年创业公寓、创业社区。探索建设海外人才离岸创业基地。

(二)加快产业转型升级，在构建现代产业体系上取得新突破

抓紧做大增量，加快推进战略性新兴产业成为支柱产业，加快规划布局未来产业。不断优化存量，加快推进传统制造业和服务业向中高端转型升级。

促进工业在稳增长中调结构。推进100个重大工业项目建设。投资50亿元以上项目开工、投产各2个，新引进5个；投资30亿元以上项目开工、投产各5个；投资10-30亿元项目开工、投产各10个。提升工业园区配套功能，开展智慧园区创建试点，实施“四大工程”和“企业上云”计划，在传统领域试点建设智能工厂、数字化车间、产业大数据云计算服务平台。出台实施领军企业计划，围绕战略性新兴产业细分领域、现有支柱产业重点领域，选择龙头企业，进行集中扶持。大力实施“小巨人”计划，制定成长路线图。创建全国质量强市示范城市。规模以上工业增加值增长10%。

培育战略性新兴产业新优势。聚焦重点、聚集资源、聚合政策，做大做强信息技术、生命健康、智能制造等新兴产业规模。争取国家集成电路基金支持，启动国家存储器基地建设。加快华星光电LTPS 6代线“点亮”，加快天马显示面板新项目、联想摩托罗拉全球产业园、海尔创新产业园建设。加快推进部分国际品牌和比亚迪、扬子江、金龙等新能源汽车项目。推进新型航天技术产业基地、格力智能装备产业园等项目开工。积极引进新兴产业龙头企业，做大全市战略性新兴产业发展基金规模。高新技术产业产值增长15%，新增高新技术企业200家。

加快发展现代服务业。分层落实、分类优化总部经济政策，广泛吸引国内外大企业、大集团来汉设立区域总部、研发中心、财务中心。引进金融机构8家，新增地方法人金融机构1家，推动10家企业在境内外上市。鼓励规范民间金融发展，防范化解金融风险。壮大工程设计产业，申报世界设计之都。发展城市配送物流，积极申报国家多式联运示范工程。改善武汉国际博览中心、武汉国际会展中心等场馆配套设施，培育、引进知名会展品牌。启动实施“两江四岸”旅游功能提升三年行动计划，加快江汉朝宗文化旅游景区等项目建设。推进“武汉·中国宝谷”建设。加快传统商贸业提档升级，支持企业开展电子商务应用，大力发展垂直电商、跨境电商等新业态，培育新型大宗商品和电子交易市场。

深入实施“互联网+”产业创新工程。加快“云端武汉”系列工程建设，推广 “智慧城市”示范项目。推动移动互联网、云计算、大数据、物联网等与制造业、服务业融合发展，培育壮大工业互联网、互联网金融等新业态。

(三)遵循城市发展规律、保持建设势头，在提升城市综合功能、改善城市品质上取得新突破

用后现代理念，推动城市规划由指导建设向规范建设与保护生态并重转变，城市建设由打造功能向提升功能与改善品质并重转变，城市管理由传统主导向社会共治与智慧现代并重转变。

强化规划法定作用。加快编制新一轮城市总体规划、土地利用总体规划、综合交通规划、主体功能区规划。抓紧编制全域生态保护规划，下决心划定全市生态边界；抓紧编制全域城镇体系规划，下决心划定全市城镇开发边界；抓紧推进主城控制性详细规划全覆盖，下决心锁定主城建设拓展边界；抓紧编制全市历史文化遗存保护规划，下决心划定历史文化保护边界。

推进铁水公空重大项目建设。做好新一轮《武汉铁路枢纽总图规划》编审工作，推动一批重大项目进入国家“十三五”铁路发展规划和综合交通网中长期发展规划。支持武汉至孝感城际铁路开通运营。建成天河机场三期，依托空港，加快“空铁公”枢纽建设，实现武深高速武汉段全线贯通。加快阳逻集装箱港铁水联运、花山码头二期、金口港区二期、汉南港项目建设，提升武汉新港集疏运功能，依托水港，加快“水铁公”枢纽建设，推动长江中游航运中心建设实现重大突破。

完善市域快速交通体系。建成地铁6号线一期、机场线，实现城铁、地铁、航空无缝对接、快速换乘。开工建设8号线二期、11号线武昌段，同时推进13条地铁线建设，编制申报第四轮轨道交通建设规划。完善三镇骨干路网，建成四环线西段等项目，完成雄楚大街、东风大道地面段和中山大道等改造。加快推进杨泗港长江大桥、沌口长江大桥、青山长江大桥、月湖桥(扩建)、汉江大道、四环线南段北段等项目。

优化城乡生态环境。提升城市主次干道和三环线内44座立交匝道绿化水平，建成东湖绿道，加快建设环汉口绿道。开工建设东风大道绿道、九真山绿道，新建绿道300公里。启动府河生态绿楔工程。造林绿化2.63万亩。制订实施水生态文明建设规划。加快治理马影河、沙河等河流和龙阳湖、墨水湖、南湖、汤逊湖等重点湖泊，全市湖泊水质保持稳定、逐步改善。新改扩建15个临江、环湖、沿河公园。完成汉西、南太子湖、黄家湖等污水处理厂改扩建，新建污水管网100公里。

深化“城管革命”。着力在精细、标准、规范上下功夫。推动城管执法重心下移。提高环卫机械化作业水平。开展户外广告、门店牌匾、指示牌、“城市家具”、城乡结合部等专项整治工作。强化住宅小区违建整治和重点工程周边违建管控。源头治理出土工地和渣土运输车辆。制订出台全市渣土消纳场建设及资源利用整体规划。加强城建工程文明施工管理。提高供水、供气、供电等公用事业服务水平。

(四)深化改革扩大开放，在增强城市发展动力活力上取得新突破

在重要领域和关键环节推出一批重大改革举措，同时，以开放促改革、促创新、促发展。

着力加强供给侧结构性改革。支持企业技术改造和设备更新。开展降本增效专项行动，千方百计降低企业制度性、人工、税费、社会保险、财务、电力、物流等方面成本，有效缓解PPI持续走低问题。减项目、降标准，严格落实涉企收费清单，做到清单之外无收费。积极稳妥推进“僵尸企业”兼并重组。进一步研究财政、金融、土地等系列政策措施，促进房地产业健康较快增长。

深化行政体制改革。探索设立区综合行政审批机构。实施财政专项资金管理改革，创新专项资金设立、使用和管理方式。清理规范行政审批中介服务。推进事业单位管养分离改革，实施公车改革方案，建立机关事业单位养老保险制度。

激发各类市场主体活力。制定实施国有企业改革系列办法，加快推进市水务集团整体上市、省客运集团上市工作。出台民间资本投资负面清单，在公共服务领域，新推出一批面向民间资本公开招标的重大项目，积极推进PPP建设模式，拓展投融资渠道。

加快发展开放型经济。创新招商方式，加大引进力度，力争招商引资总额增长20%，实际利用外资增长16%。积极争取开展跨境电子商务试点，申报国家服务贸易创新发展试点城市，外贸出口增长10%。引导企业争取承担“一带一路”建设项目，支持企业在国外建设武汉产业园，开展产能合作。

提升城市开放功能。新开通国际及地区航线3条，发展国际全货机航线。中欧(武汉)国际货运班列延伸至法国。完善近海近洋航线网络，增强中西部“出海口”功能。争取设立武汉新港空港综合保税区，力争湖北(武汉)内陆自贸区、武汉长江新区尽早获批。做好促进俄罗斯总领事馆设立和美国总领事馆开办签证工作。新增国际友好城市和国际友好交流城市10个。积极融入长江经济带发展，扩大与长江中游城市在交通基础设施、产业链配套、政府公共服务等领域合作。

(五)加快转变农业发展方式，在创建国家现代农业示范区上取得新突破

发展现代高效生态农业，大力推广新品种、新技术、新模式，提升农业发展质量效益。

大力发展现代都市农业。新增农业标准化生产示范基地1.5万亩。加快建设7个现代农业示范园区。鼓励支持新型市场主体参与“菜篮子”工程建设。推广适用新型农业机械1.5万台(套)。实现农产品加工产值2680亿元。推进中国种都建设。加快发展智慧农业、循环生态农业，扎实开展“互联网+农业”行动，大力发展农产品电子商务平台，支持加快建设300个村级电子商务综合服务站。

健全现代农业经营体系。以土地流转为基础，积极发展多种形式适度规模经营，加快培育农业企业，新增新型农业经营主体超过1000个。支持新城区创建国家和省农产品质量安全示范区。深入推进供销合作社综合改革和农垦改革发展。做大做强农村产权交易市场。

建设美丽乡村。开展农村垃圾专项治理，加大农村污水处理和改厕力度，全市行政村环境卫生全部达标。扎实推进一批美丽乡村示范村建设。全面完成农村排灌港渠整治工程，切实解决农田灌溉“最后一公里”问题。

(六)加强社会建设，在保障和改善民生上取得新突破

在推进经济、城市升级的同时，更加注重民生升级，让市民享受到更多的发展成果。

强化就业和社会保障工作。城镇新增就业16万人，农村劳动力转移就业5万人，新增创业2.5万人。深化医疗保险制度改革，完善居民、个体从业人员、农民工等群体养老保险政策。社会保险净增参保35万人次。提高企业退休人员、机关事业单位退休人员基本养老金。实施棚户区改造5.2万户。确保完成省下达的住房保障目标任务。加强社会救助救济工作，继续提高城乡低保标准。

提升公共服务质量。促进教育公平优质发展，全面实施义务教育学区制管理，打造一批高水平职教实习实训基地，支持在汉高校建设世界一流大学、一流学科。提高卫生计生服务水平，稳步开展公立医院综合改革，确保医改“惠民强医”，提高基层卫生服务能力，积极推进分级诊疗试点工作，鼓励兴办国际性特色医疗机构。

繁荣发展文体事业。超前做好迎接建国70周年工作，建设一批标志性重大工程。建成中共中央机关旧址纪念馆，开工建设盘龙城博物馆。全面启动2019年第七届世界军人运动会场馆、谌家矶足球超级杯等体育场馆建设。办好首届武汉国际马拉松赛、第三届WTA武汉网球公开赛、7·16全民游泳健身周等品牌赛事。

创新社会治理。落实创新社会治理加强基层建设“1+10”系列改革举措。持续推进社区“两委”成员依法进入业主委员会，完善社区居委会、业主委员会、物业服务企业“三方联动”工作机制，提升物业服务和社区治理水平。启动“七五”普法。严厉打击“两抢一盗”等违法犯罪行为。全面落实“五个必须”，树立“隐患就是事故”的观念，及时查处整改各类安全生产隐患。创建国家食品安全城市。积极服务军队调整改革，深化军民融合发展，争创全国双拥模范城“六连冠”。

“天地之大，黎元为先。”今年，我们将围绕广大市民关切的问题，继续办好十件实事。

1、缓解交通拥堵。完善智慧交通系统，加强停车行车诱导，实现交通路况“一月一通报、一周一预测、天天时时知”。综合整治60处交通堵点和90处路段安全隐患。建成微循环道路100条。增加主次干道人行横道灯2000套、车行信号灯1500套，设置立交匝道和弯道主动发光警示标志175处，更新、完善4万个路内停车泊位标识、标线。新增停车泊位2万个。

2、改善空气质量。实施“拥抱蓝天”行动方案。全市煤炭消费总量下降2.6%。在中心城区城建工地，安装扬尘污染在线监控设施100套。推动100个建设工程工地安装喷洒降尘设施。整治机场、火车站、长途汽车站等地带的渣土、扬尘、垃圾污染。完成餐饮油烟治理改造2.5万家。全面供应国V车用汽柴油，推广新能源汽车5000辆。

3、减少城市渍水。开展海绵城市试点，实施青山、四新示范区150个海绵化改造项目。新建排水管网100公里，完成汉钢西、后湖三期泵站改造建设及21号公路尾水明渠改扩建等6个排水项目，启动11个排水项目建设。汉阳王家湾、琴台大道、汉口沿江、武昌车辆厂、秦园路等地区渍水问题得到明显缓解。

4、关爱残疾人和重大疾病儿童。建成30家精神障碍患者康复机构。全市重度听力、言语残疾人免费乘坐公交车、轻轨、地铁和轮渡。为全市婴儿免费实施先天性心脏病筛查，对需要手术治疗的患儿给予2万元的医疗费用补助。

5、实施精准扶贫，改善老旧社区环境。聚焦精准扶贫，开展好第六轮“三万”活动，实现77个贫困村脱贫出列、43369名农村贫困人口脱贫销号。建设青少年寒暑假期日间托管室150个。连片整治133个老旧社区、60条城市道路架空管线。

6、改善就学就医条件。改造升级全市660所中小学校园安全视频系统。继续为新入学的全日制大学生每人赠送一本市民手册和一张“武汉通”公交乘车卡。提档升级10家社区卫生服务中心、15家乡镇卫生院。

7、强化食品安全保障。提升1000家小餐饮店食品安全等级。在500家餐饮服务单位推行“明厨亮灶”。开展食品安全抽样检测3万批次，每月公布抽检信息。

8、方便市民信息消费。在中心城区，实施“百兆入户”工程，80%以上宽带用户接入能力达到100兆。解决1000个公共区域公益免费无线局域网热点网络不畅、网速慢、易掉线的问题。在新城区，实现光纤“村村通”。

9、推进文体惠民。为498支基层群众文艺团队配置活动器材。在35家游泳场所，为中小学生提供20天免费暑期游泳。对4000名青少年开展游泳、足球、网球免费培训。

10、扶持创新创业。重点支持高校集中区域建设2个以上创新园区；择优支持新建“创谷”，新增50家众创空间，支持创新园区和平台为创客提供“拎包入住、百兆上桌、代理中介”等服务。帮助2万名创新创业者创业。新增大学生创新创业企业2000家以上。

(七)围绕创新务实、担当高效，在政府自身建设上取得新突破

“为政贵在行,以实则治,以文则不治。”我们将巩固“三严三实”专题教育成果，加强自身建设，激励干事创业，不断开创政府工作新局面。

严格依法行政。认真落实市人大常委会立法工作计划。完善依法科学民主决策机制，落实政府重大行政决策程序规定。严格行政执法责任制和执法过错责任追究制。完善大城管综合执法体制，深化市场监管执法体制改革。推进行政复议规范化建设。加强行政应诉工作。自觉接受市人大及其常委会的法律监督、工作监督和市政协的民主监督。切实办理好人大议案、代表建议和政协建议案、提案。

提升行政效能。运用法治思维、市场思维、互联网思维，着力提高深化改革、推动发展、化解矛盾、维护稳定的能力。加快“云端武汉·政务”建设，打造政务数据资源共享平台，试点建设移动互联政务办公系统。在市、区政务服务中心试行“前台统一受理、后台分类审批、统一窗口出件”模式。优化武汉市民之家办事流程、服务流程，线上申办事项比例达90%以上。进一步精简会议和文件，办有效的事，开务实的会，发管用的文。

强化责任担当。严格绩效管理，健全与五大发展理念相一致的目标考核奖惩评价体系。对外公开责任，对内闭环责任，构建严密的责任链。深化治庸问责工作，用好电视问政等平台，及时解决群众反映的热点问题，严肃查处庸懒散和行政不作为、慢作为、乱作为问题。探索建立改革创新免责机制，激励干事创业。

加强廉政建设。坚持“力度统一论”，严格落实党风廉政建设主体责任和“一岗双责”。切实加强工程建设、土地出让、政府采购、产权交易等重点领域监管，对各类违法违纪案件始终做到零容忍、严惩治，坚决纠正损害群众利益的不正之风。我们将以抓铁有痕的精神，持之以恒抓好法治政府和服务型政府建设。

各位代表：

鲲鹏展翅凌万里，逐梦扬帆再起航。“十三五”蓝图已经绘就，只要我们接续奋斗、坚定前行，梦想总是可以实现的。让我们紧密团结在以习近平同志为总书记的党中央周围，在省委、省政府和市委的坚强领导下，开拓创新、只争朝夕，谱写建设国家中心城市、复兴大武汉的壮丽篇章!
